# The Effect of β-Lactam Antibiotics on the Evolution of Ceftazidime/Avibactam and Cefiderocol Resistance in KPC-Producing Klebsiella pneumoniae

**DOI:** 10.1128/aac.01279-22

**Published:** 2023-02-16

**Authors:** Ping Zhang, Huangdu Hu, Qiucheng Shi, Long Sun, Xueqing Wu, Xiaoting Hua, Alan McNally, Yan Jiang, Yunsong Yu, Xiaoxing Du

**Affiliations:** a Department of Infectious Diseases, Sir Run Run Shaw Hospital, Zhejiang University School of Medicine, Hangzhou, China; b Key Laboratory of Microbial Technology and Bioinformatics of Zhejiang Province, Hangzhou, China; c Regional Medical Center for National Institute of Respiratory Diseases, Sir Run Run Shaw Hospital, Zhejiang University School of Medicine, Hangzhou, China; d Department of Clinical Laboratory, Hangzhou Women’s Hospital (Hangzhou Maternity and Child Health Care Hospital), Hangzhou, China; e Institute of Microbiology and Infection, College of Medical and Dental Sciences, University of Birmingham, Birmingham, United Kingdom

**Keywords:** β-lactam antibiotics, evolution, ceftazidime/avibactam, cefiderocol, *bla*_KPC-2_, amplification, mutation

## Abstract

In this study, we aimed to clarify the evolutionary trajectory of a Klebsiella pneumoniae carbapenemase (KPC)-producing Klebsiella pneumoniae (KPC-Kp) population during β-lactam antibiotic therapy. Five KPC-Kp isolates were collected from a single patient. Whole-genome sequencing and a comparative genomics analysis were performed on the isolates and all *bla*_KPC-2_-containing plasmids to predict the population evolution process. Growth competition and experimental evolution assays were conducted to reconstruct the evolutionary trajectory of the KPC-Kp population *in vitro*. Five KPC-Kp isolates (KPJCL-1 to KPJCL-5) were highly homologous, and all harbor an IncFII *bla*_KPC_-containing plasmid (pJCL-1 to pJCL-5). Although the genetic structures of these plasmids were almost identical, distinct copy numbers of the *bla*_KPC-2_ gene were detected. A single copy of *bla*_KPC-2_ was presented in pJCL-1, pJCL-2, and pJCL-5, two copies of *bla*_KPC_ (*bla*_KPC-2_ and *bla*_KPC-33_) were presented in pJCL-3, and three copies of *bla*_KPC-2_ were presented in pJCL-4. The *bla*_KPC-33_-harboring KPJCL-3 isolate presented resistance to ceftazidime-avibactam and cefiderocol. The *bla*_KPC-2_ multicopy strain KPJCL-4 had an elevated ceftazidime-avibactam MIC. The patient had been exposed to ceftazidime, meropenem, and moxalactam, after which KPJCL-3 and KPJCL-4 were isolated, which both showed a significant competitive advantage under antimicrobial pressure *in vitro*. Experimental evolution assays revealed that *bla*_KPC-2_ multicopy-containing cells were increased in the original single-copy *bla*_KPC-2_-harboring KPJCL-2 population under selection with ceftazidime, meropenem, or moxalactam, generating a low-level ceftazidime-avibactam resistance phenotype. Moreover, *bla*_KPC-2_ mutants with a G532T substitution, G820 to C825 duplication, G532A substitution, G721 to G726 deletion, and A802 to C816 duplication increased in the *bla*_KPC-2_ multicopy-containing KPJCL-4 population, generating high-level ceftazidime-avibactam resistance and reduced cefiderocol susceptibility. Ceftazidime-avibactam and cefiderocol resistance can be selected by β-lactam antibiotics other than ceftazidime-avibactam. Notably, *bla*_KPC-2_ gene amplification and mutation are important in KPC-Kp evolution under antibiotic selection.

## INTRODUCTION

Carbapenem-resistant *Enterobacteriaceae* (CRE) are listed as pathogens that urgently require new antibiotics (1). Ceftazidime-avibactam (CAZ/AVI) and cefiderocol (CFDC) are some of the most recently developed antibiotics used to treat CRE, with a high clinical success rate (2, 3). However, the emergence of CAZ/AVI and CFDC resistance in CRE has been reported.

Resistance to CAZ/AVI in Klebsiella pneumoniae carbapenemase (KPC)-producing Klebsiella pneumoniae (KPC-Kp) is associated mainly with *bla*_KPC-2_ or *bla*_KPC-3_ mutation and *bla*_KPC_ overexpression (4). *bla*_KPC_ overexpression is often accompanied by changes in membrane permeability or increased efflux pump expression to yield low-level CAZ/AVI resistance (5, 6). High-level CAZ/AVI resistance is often related to *bla*_KPC-2_ and *bla*_KPC-3_ mutations. The most common mutation is the G532T substitution, which leads to the D179Y amino acid change in KPC-3 (named KPC-31) and KPC-2 (named KPC-33). Most KPC variants were selected after exposure to CAZ/AVI (7), and some of the KPC variants conferred cross-resistance to CAZ/AVI and CFDC (8, 9), which is a clinical concern in the application of antibiotics.

Although CAZ/AVI-resistant isolates are associated with CAZ/AVI selection pressure, data showed that 33% of cases had no previous CAZ/AVI exposure (7). Here, we analyzed five homologous KPC-Kp isolates from a single patient. After exposure to ceftazidime, meropenem, and moxalactam, the KPC-Kp population showed a temporal pattern of evolution to high-level CAZ/AVI and CFDC resistance through *bla*_KPC-2_ gene amplification and mutation. We reconstructed the evolutionary pathway *in vitro* using experimental evolution assays.

## RESULTS

### Patient and isolates.

KPJCL-1 to KPJCL-5 were sequentially isolated from a 56-year-old male patient who was admitted to the intensive care unit (ICU) due to cerebral hemorrhage. KPJCL-1 was isolated from urine on hospitalization day 55 and diagnosed as colonization. A scrotal abscess developed on hospitalization day 91, and KPJCL-2 was isolated. Although the patient underwent scrotal abscess incision and drainage, the abscess was sustained, and a fistulous tract appeared between the abscess and the urinary tract. KPJCL-3 was isolated from the abscess on hospitalization day 142, and KPJCL-4 was isolated from urine on hospitalization day 156. Before the isolation of KPJCL-3 and KPJCL-4, the patient was treated with moxalactam for 13 days (1.0 g once a day [q.d.], days 92 to 104), meropenem for 20 days (1.0 g every 12 h, days 104 to 106; 0.5 g every 12 h, days 106 to 123), and ceftazidime for 16 days (1.0 g every 12 h, days 123 to 138). The scrotal abscess persisted, and KPJCL-5 was sampled from the abscess on hospitalization day 196. The patient died on hospitalization day 220 due to septic shock (Fig. S1 in the supplemental material).

### Phenotypic and genotypic characteristics of the KPC-Kp isolates.

**FIG 1:**
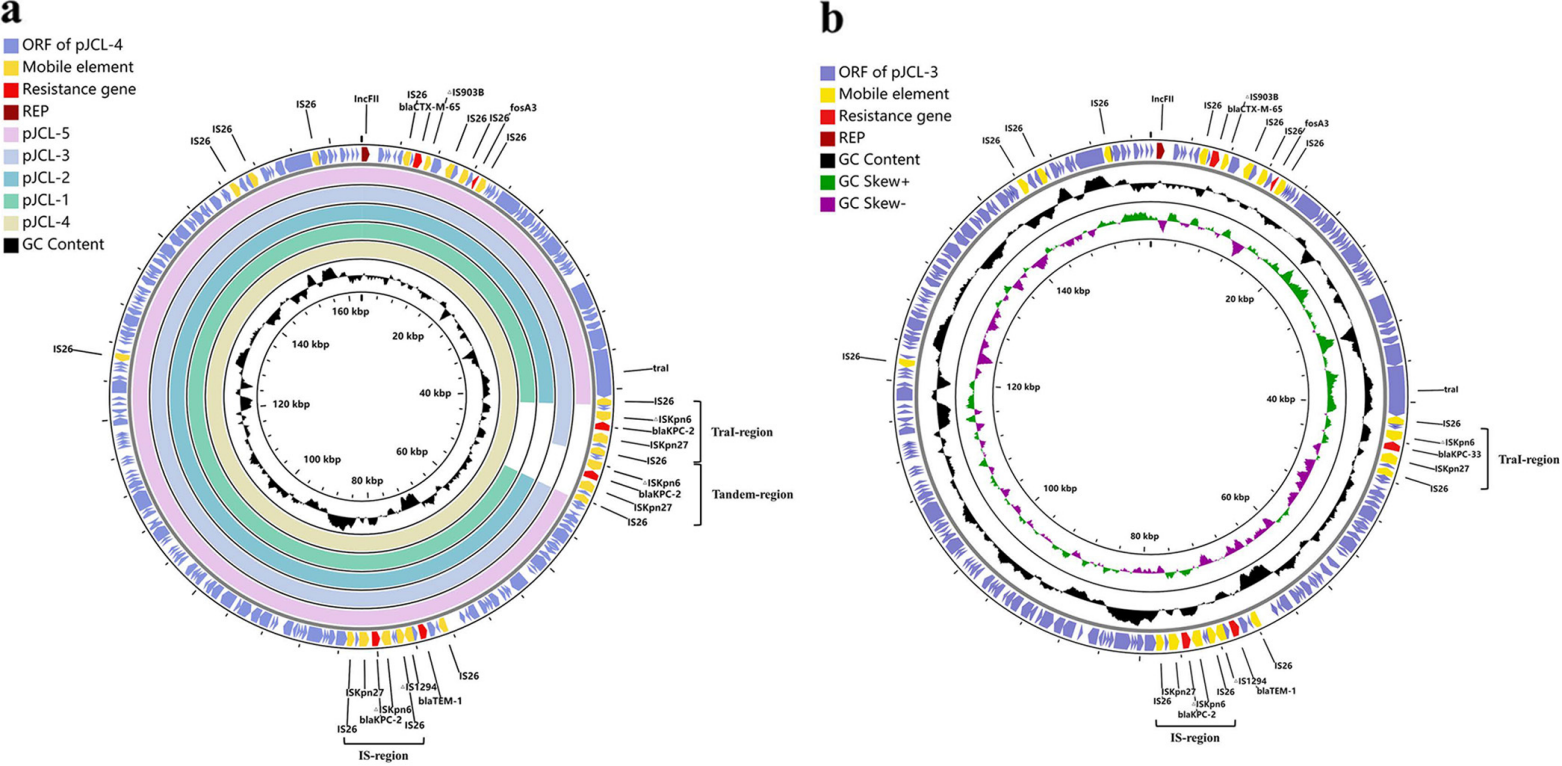
Gene maps of *bla*_KPC-2_-containing IncFII plasmids. (a) pJCL-4 was used as a reference. The outside circle represents open reading frames (ORFs) of pJCL-4. The inner circles are BLAST results for pJCL-5, pJCL-3, pJCL-2, pJCL-1, and pJCL-4 and the GC content. Antibiotic resistance genes and their surrounding mobile elements are colored in red and yellow. The three *bla*_KPC-2_ regions are named the TraI, tandem, and IS regions. Plasmid sequences were compared using the CGView server. REP, replicon. (b) Characteristics of pJCL-3. The outside circle represents ORFs of pJCL-3. The inner circles are the GC content and GC skew. Antibiotic resistance genes and their surrounding mobile elements are colored red and yellow, respectively. In the TraI region, *bla*_KPC-2_ was mutated to *bla*_KPC-33_.

The five KPC producers belong to sequence type 11 (ST11). A comparative analysis showed that the isolates differed by 3 to 7 single nucleotide polymorphisms (SNPs) (Fig. S2), while the drug susceptibility phenotypes differed (Table 1). KPJCL-1, KPJCL-2, and KPJCL-5 were sensitive to CAZ/AVI and CFDC. KPJCL-3 was resistant to CAZ/AVI and CFDC and had an elevated ceftazidime MIC compared to other isolates. KPJCL-4 exhibited an increase in MICs for antibiotics, including CAZ/AVI, ceftazidime, meropenem, and moxalactam. All isolates harbored an IncFII *bla*_KPC_-containing plasmid (pJCL-1 to pJCL-5), and the plasmid sizes were 160,120, 160,127, 157,880, 163,236, and 160,001 bp, respectively. A single copy of *bla*_KPC-2_ was present in pJCL-1, pJCL-2, and pJCL-5, two copies of *bla*_KPC_ (*bla*_KPC-2_ and *bla*_KPC-33_) were present in pJCL-3, and three copies of *bla*_KPC-2_ were present in pJCL-4 (Fig. 1). The five *bla*_KPC_-containing plasmids have an identical *bla*_KPC-2_ region (named the IS region). pJCL-3 and pJCL-4 contain extra *bla*_KPC_ copies (TraI region); moreover, pJCL-4 contains a tandem repeat of *bla*_KPC-2_ (tandem region) next to the TraI region compared to pJCL-3. Cloning experiments showed that KPC-33 conferred reduced susceptibility to ceftazidime, CAZ/AVI, and CFDC compared to KPC-2 (Table S2).

**TABLE 1:** Characteristics of KPC-Kp isolates described in the present study

Isolates	Isolation day	Source	Carbapenemase	*bla*_KPC_ copy no.^*a*^	MIC (mg/L)^*b*^									
					IMP	MEM	CAZ/AVI	CFDC	CAZ	MOX	TGC	COL	LEV	ST
KPJCL-1	55	Urine	KPC-2	Single copy	64	256	4	4	256	512	0.5	0.25	16	11
KPJCL-2	91	Abscess	KPC-2	Single copy	32	128	4	4	128	512	0.5	0.25	32	11
KPJCL-3	142	Abscess	KPC-2, KPC-33	Dual copy	32	256	256	>32	4,096	2,048	0.5	0.25	64	11
KPJCL-4	156	Urine	KPC-2	Triple copy	128	512	8	4	1,024	4,096	0.5	0.25	64	11
KPJCL-5	196	Abscess	KPC-2	Single copy	32	128	4	4	128	512	0.5	0.25	64	11
ATCC 25922	-^*c*^	-^*c*^	-^*c*^	-^*c*^	0.125	0.016	0.125	0.25	0.25	0.25	0.03	0.25	0.016	-^*c*^

a *bla*_KPC_ copy number: *bla*_KPC_ copy number relative to the *bla*_KPC_-containing plasmid.

b IMP, imipenem; MEM, meropenem; CAZ/AVI, ceftazidime-avibactam; CFDC, cefiderocol; CAZ, ceftazidime; MOX, moxalactam; TGC, tigecycline; COL, colistin; LEV, levofloxacin; ST, sequence type.

c Not applicable.

### Growth and competition between *bla*_KPC-2_ single- and multicopy strains.

**FIG 2:**
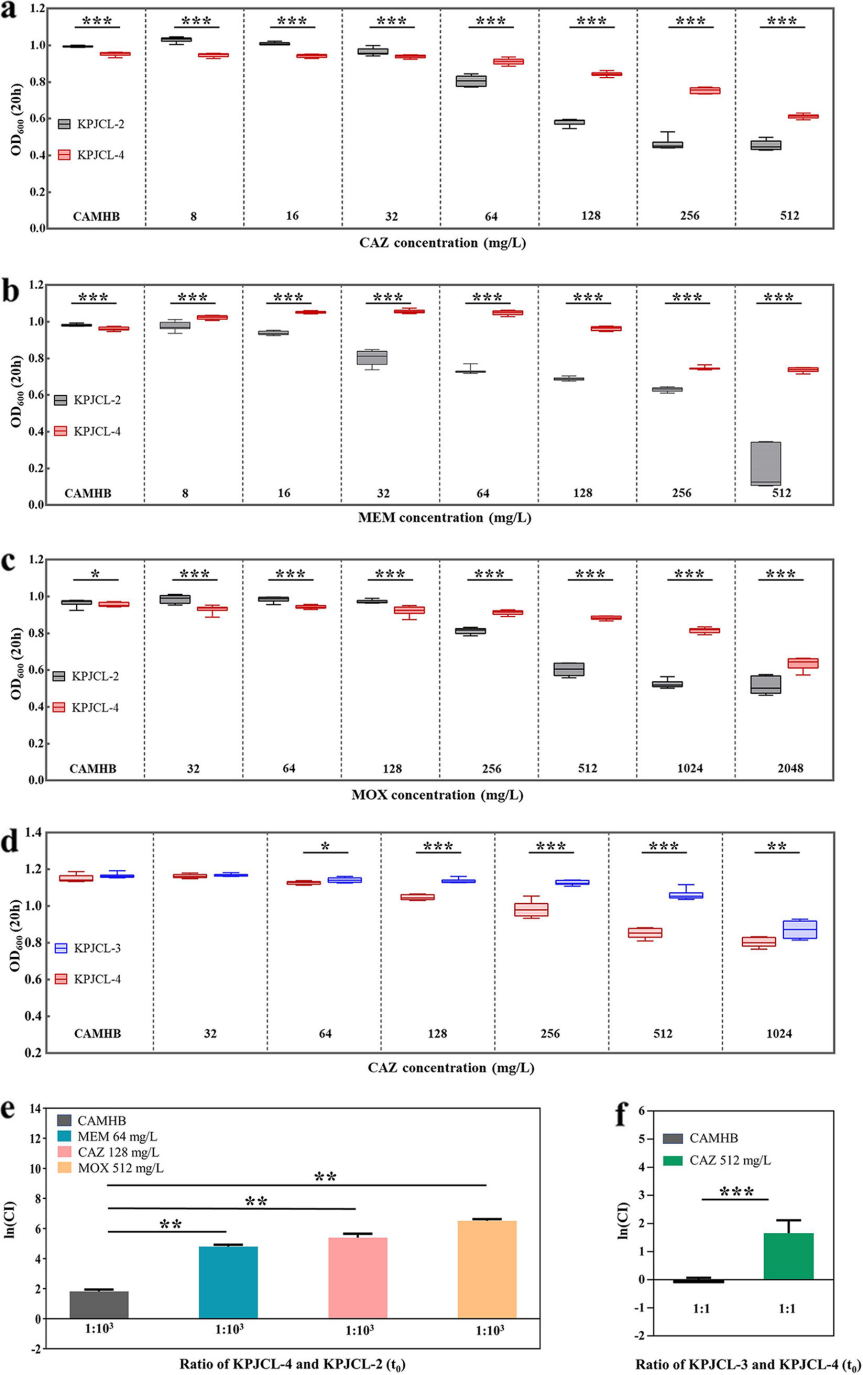
Growth assessment and competition assays. (a to c) Difference in growth between KPJCL-2 and KPJCL-4 under ceftazidime (a), meropenem (b), and moxalactam (c) pressure; CAZ, ceftazidime; MEM, meropenem; MOX, moxalactam. The greatest growth advantage of KPJCL-4 was observed at concentrations of 128 mg/L ceftazidime, 64 mg/L meropenem, and 512 mg/L moxalactam. (d) Difference in the growth of KPJCL-3 and KPJCL-4 under ceftazidime pressure. The greatest growth advantage of KPJCL-3 was observed at a ceftazidime concentration of 512 mg/L. (e) Growth competition of KPJCL-2 and KPJCL-4 under a ceftazidime concentration of 128 mg/L, a meropenem concentration of 64 mg/L, and a moxalactam concentration of 128 mg/L. (f) Growth competition of KPJCL-3 and KPJCL-4 in the presence of a ceftazidime concentration of 512 mg/L. CI = (KPJCL-4/KPJCL-2)*_t_*_24_/(KPJCL-4/KPJCL-2)*_t_*_0_, where an ln (CI) of <0 indicates that KPJCL-2 has an advantage, and an ln (CI) of >0 indicates that KPJCL-4 has an advantage (e). CI = (KPJCL-3/KPJCL-4)*_t_*_24_/(KPJCL-3/KPJCL-4)*_t_*_0_, where an ln (CI) of <0 indicates that KPJCL-4 has an advantage, and an ln (CI) of >0 indicates that KPJCL-3 has advantage (f). Data are presented as the medians (maximum to minimum values) in a to d and as the means ± SD in e and f; *, *P* < 0.05; **, *P* < 0.01; ***, *P* < 0.001. Data were analyzed by a two-sided Mann-Whitney *U* test.

KPJCL-2 (single copy) and KPJCL-4 (multicopy) were chosen as a pair of experimental strains to verify that *bla*_KPC-2_ multicopy strains were more fit than *bla*_KPC-2_ single-copy strains under ceftazidime, meropenem, and moxalactam pressure. KPJCL-4 was less fit than KPJCL-2 in cation-adjusted Mueller-Hinton broth (CAMHB) but had a growth advantage under ceftazidime, meropenem, and moxalactam pressure. The greatest growth advantage of KPJCL-4 was observed in the presence of antibiotic concentrations at the MICs or sub-MICs of KPJCL-2, namely, 128 mg/L ceftazidime, 64 mg/L meropenem, and 512 mg/L moxalactam (Fig. 2a to c; Fig. S3 to S5). KPJCL-4 showed a significant competitive advantage at an inoculum proportion of 1:1 under ceftazidime (128 mg/L), meropenem (64 mg/L), and moxalactam (512 mg/L) pressure, and the ln (competition index [CI]) values could not be calculated; therefore, we adjusted the initial coculture ratio to 1:10^3^ (KPJCL-4:KPJCL-2). A significant competitive advantage for KPJCL-4 was observed (ln [CI] values were 4.79 ± 0.13, 5.39 ± 0.26, and 6.52 ± 0.10 under ceftazidime, meropenem, and moxalactam pressure, respectively) (Fig. 2e).

### Growth and competition between *bla*_KPC-2_ multicopy and *bla*_KPC-33_ strains.

KPJCL-3 (*bla*_KPC-33_ harboring) and KPJCL-4 (multicopy) were chosen as a pair of experimental strains. KPJCL-3 showed a growth advantage under ceftazidime pressure, and dominance was greatest at 512 mg/L (Fig. 2d; Fig. S6). At an inoculum proportion of 1:1, the two isolates exhibited comparable growth in CAMHB (ln [CI] = −0.11 ± 0.17), while a significant competitive advantage for KPJCL-3 was observed under pressure with 512 mg/L ceftazidime (ln [CI] = 1.66 ± 0.46) (Fig. 2f).

### Experimental evolution of KPJCL-2 under ceftazidime, meropenem, and moxalactam pressure.

**FIG 3:**
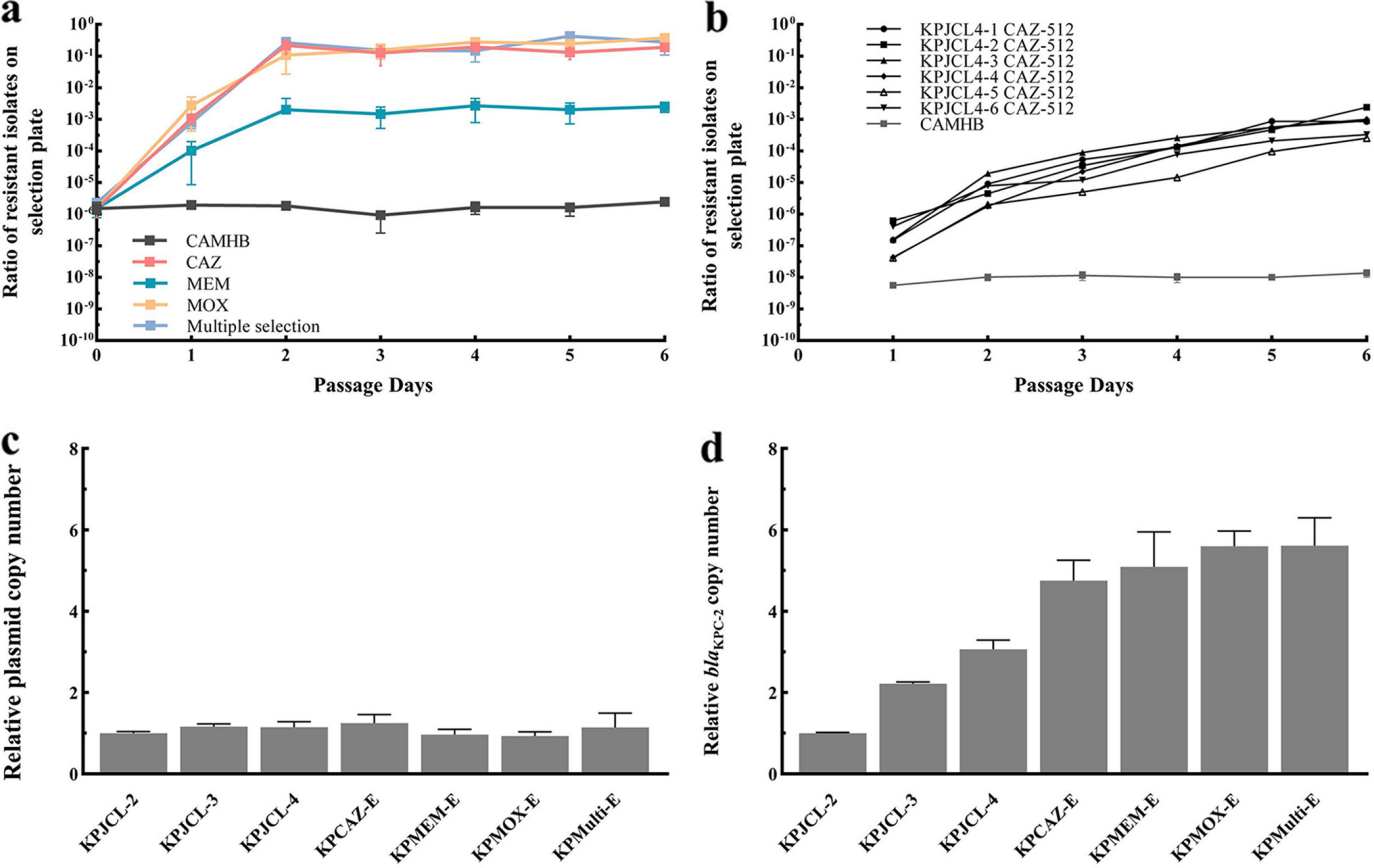
Experimental evolution of resistance. (a) *In vitro* selection and enrichment of *bla*_KPC-2_ multicopy strains. The proportion of colonies detected over time in CAMHB (6 days) or in the presence of a ceftazidime concentration of 128 mg/L (6 days), a meropenem concentration of 64 mg/L (6 days), a moxalactam concentration of 128 mg/L (6 days), and multiple selection (moxalactam, meropenem, and ceftazidime each lasting for 2 days). Data are presented as the means ± SD. (b) *In vitro* selection and enrichment of *bla*_KPC-2_ mutant strains. The proportion of *bla*_KPC-2_ mutant isolates obtained over time in CAMHB and after treatment with a ceftazidime concentration of 512 mg/L is shown. Six populations that developed an increased proportion were named KPJCL4-1 to KPJCL4-6. No enrichment of the population was observed in CAMHB, and the curve shows the mean proportions ± SD from 12 populations. (c) Relative *bla*_KPC-2_-containing plasmid copy number (mean ± SD). (d) Relative *bla*_KPC-2_ copy number (*bla*_KPC_ copy number relative to *bla*_KPC_-containing plasmid). Data are presented as the means ± SD.

The *bla*_KPC-2_ amplification frequency was 5.96 × 10^−7^ in the KPJCL-2 population (Fig. S7). Under selection pressure with ceftazidime, meropenem, or moxalactam, the frequency of *bla*_KPC-2_ multicopy cells in the KPJCL-2 population increased substantially, reaching 1.9 × 10^−1^, 2.52 × 10^−3^, and 3.75 × 10^−1^ on day 6, respectively. KPJCL-2 cells were passaged sequentially in moxalactam, meropenem, and then ceftazidime (2 days for each) to simulate the sequential use of antibiotics in the clinic. The frequency of *bla*_KPC-2_ multicopy cells was 2.78 × 10^−1^ at the end of passaging (Fig. 3a).

### An elevated *bla*_KPC-2_ copy number contributes to low-level CAZ/AVI resistance.

The relative *bla*_KPC-2_ copy number (copy number relative to the *bla*_KPC-2_-containing plasmid) was 4.750 ± 0.501 in the ceftazidime-selected subpopulation (KPCAZ-E), 5.089 ± 0.860 in the meropenem-selected subpopulation (KPMEN-E), 5.598 ± 0.373 in the moxalactam-selected subpopulation (KPMOX-E), and 5.615 ± 0.681 in the subpopulation exposed to all three antibiotics (KPMulti-E). The relative *bla*_KPC_-containing plasmid copy numbers were unchanged among the isolates (Fig. 3c and d). The selected *bla*_KPC-2_ multicopy subpopulation exhibited elevated CAZ/AVI (16 mg/L), ceftazidime (512 mg/L), meropenem (512 mg/L), and moxalactam (2,048 mg/L) MICs compared to KPJCL-2 but had similar CFDC MICs as KPJCL-2 (Table S3).

### Experimental evolution of KPJCL-4 under ceftazidime pressure.

KPJCL-4 has the closest genetic relationship with KPJCL-3, and we wanted to observe the distribution of mutations occurring in the three *bla*_KPC-2_ regions (the TraI, IS, and tandem regions); therefore, KPJCL-4 was chosen as the target population for evolution. The *bla*_KPC-2_ G532T mutation frequency in KPJCL-4 was 6.45 × 10^−10^, which was much lower than the *bla*_KPC-2_ gene amplification frequency (Fig. S7). Of the 18 lineages cultured with a ceftazidime concentration of 512 mg/L, six populations developed increased frequency of resistant colonies on antibiotic plates, from an average frequency of 2.38 × 10^−7^ after 24 h of exposure to 9.57 × 10^−4^ after 6 days of exposure. No increase in the frequency of resistance subgroups was observed in control lineages in CAMHB (Fig. 3b).

### The *bla*_KPC-2_ mutation contributes to high-level CAZ/AVI and CFDC resistance.

KPC-2 variants were identified in all 6 evolved subpopulations (KPJCL4-1 to KPJCL4-6). Two variants were observed in KPJCL4-1, colonies with a KPC-2 D179Y substitution (KPC-33) or a E275 to A276 duplication (novel). KPC-33 was also detected in KPJCL4-2 and KPJCL4-3. The D179N variant was identified in KPJCL4-4, the G242-T243del variant (KPC-14) was found in KPJCL4-5, and the K269-H273dup variant (novel) was identified in KPJCL4-6. The *bla*_KPC-2_ mutation occurred randomly among the three regions. These evolved subgroups are highly resistant to CAZ/AVI with increased ceftazidime MICs compared to KPJCL-4 (Table 2). All mutants displayed intermediate or were resistant to CFDC.

**TABLE 2:** Characteristics of *bla*_KPC-2_ mutant subgroups from the *in vitro* evolution experiment

Isolate	*bla*_KPC_ variant	KPC-2 variant	Mutation^*a*^			MIC (mg/L)^*a*^			
			TraI region	IS region	Tandem region	CAZ/AVI	CFDC	IMP	CAZ
KPJCL-4	*bla*_KPC-2_	KPC-2	−	−	−	8	4	128	1,024
KPJCL4-1	*bla*_KPC-2_ G532T	KPC-33	−	−	+	256	>32	128	4,096
	*bla*_KPC-2_ 820-825dup	E275-A276dup	−	+	−	256	8	128	2,048
KPJCL4-2^*b*^	*bla*_KPC-2_ G532T	KPC-33	+	−	+	256	>32	128	4,096
	*bla*_KPC-2_ G532T	KPC-33	−	+	+	256	>32	128	8,192
KPJCL4-3	*bla*_KPC-2_ G532T	KPC-33	+	−	−	256	>32	128	4,096
KPJCL4-4^*c*^	*bla*_KPC-2_ G532A	D179N	+	−	+	64	16	64	4,096
	*bla*_KPC-2_ G532A	D179N	+	+	+	128	>32	2	4,096
KPJCL4-5	*bla*_KPC-2_ 721-726del	KPC-14	−	+	−	512	>32	128	8,192
KPJCL4-6	*bla*_KPC-2_ 802-816dup	K269-H273dup	−	−	+	256	8	128	4,096

a CAZ/AVI, ceftazidime-avibactam; IMP, imipenem; CAZ, ceftazidime; +, mutation occurred; −, no mutation occurred.

b Some of the colonies in KPJCL4-2 had mutations in both the TraI region and tandem region, and some had mutations in both the IS region and tandem region.

c Some of the colonies in KPJCL4-4 had mutations in both the TraI region and tandem region, and some had mutations in all three regions.

## DISCUSSION

In the present study, we demonstrate that clinical commonly used β-lactam antibiotics can select for CAZ/AVI and CFDC resistance. Using clinical data, drug susceptibility phenotypes, KPC-Kp genotypes, growth competition assays, and experimental evolution assays, we reconstructed the evolutionary trajectory of CAZ/AVI and CFDC resistance in KPC-Kp observed *in vivo*.

Based on the genomic data and the temporal pattern of isolation, we hypothesized that the evolutionary trajectory of the bacterial population developed from a *bla*_KPC-2_ single-copy population to a *bla*_KPC-2_ multicopy population and then a *bla*_KPC_ G532T mutant population (as summarized in Fig. S8 in the supplemental material). Amplification of *bla*_KPC-2_ via an increased copy number on a plasmid facilitates the development of antibiotic resistance. First, the increased *bla*_KPC-2_ copy number on plasmids increases bacterial tolerance to β-lactam antibiotics. Second, it increases the opportunity for mutations to arise (10). Our results showed an up to 6-fold increase in the *bla*_KPC-2_ copy number on plasmids after antibiotic selection, which provides a substantial increase in the number of targets for the occurrence of random mutations within *bla*_KPC-2_. Third, multiple copies of *bla*_KPC-2_ genes on the same plasmid with one of the *bla*_KPC-2_ mutations enabled the strain to compete against the trade-off effect of *bla*_KPC_ mutation. For the majority of reported CAZ/AVI-resistant strains, the mutation of a single copy of *bla*_KPC_ in the plasmid restores susceptibility to carbapenems (11). In this case, two copies of *bla*_KPC_ (*bla*_KPC-2_ and *bla*_KPC-33_) present in the same plasmid in pJCL-3 enable KPJCL-3 to resist both carbapenems and CAZ/AVI. Fourth, amplification can occur at much higher rates than point mutations (12, 13). The difference in the frequencies of amplification (5.96 × 10^−7^) and mutation (6.45 × 10^−10^) in this study likely explains the evolutionary trajectory of the KPC-Kp population, with gene amplification emerging first under antibiotic selection.

After repeated exposure to ceftazidime at concentrations of 512 mg/L, 6 populations developed mutated colonies with reduced susceptibility to CAZ/AVI and CFDC, and half of these resistant subpopulations had the D179Y substitution in KPC-2. In addition, other mutations associated with CAZ/AVI and CFDC resistance were identified. One of the variants is a D179N substitution in KPC-2. The D179N substitution in KPC-3 has been reported in a previous study of bacteria derived under CAZ/AVI pressure *in vitro* (11). The D179N substitution might increase the affinity between ceftazidime and the variant KPC, thereby preventing the binding of avibactam (14). Another variant is KPC-14, which was reported in a case after prolonged exposure to CAZ/AVI (15). KPC-14 does not affect the inhibitory properties of avibactam; however, it possesses a higher ceftazidime affinity and increased ceftazidime hydrolysis (16). To the best of our knowledge, the KPC-2 variants E275-A276dup and K269-H273dup we observed in our experiments have not been described thus far. The KPC-2 variants that emerged during ceftazidime exposure overlap with those selected under CAZ/AVI pressure, with mutations clustering in three regions, that is, amino acids 164 to 179 (Ω-loop), 234 to 242 (β-strand), and 263 to 277 (in the vicinity of the Ω-loop and the hinge-loop) (11, 17, 18). The tolerance to amino acid substitutions, insertions, and deletions reflects the evolutionary diversity of *bla*_KPC_, which is a challenge for the clinical application of CAZ/AVI and CFDC.

The concentrations of ceftazidime, meropenem, and moxalactam that we tested *in vitro* are not readily achieved in most infection sites. However, KPC-Kp strains were isolated from urine or a scrotal abscess in this study, and a fistulous tract appeared between the abscess and the urinary tract in the patient; thus, the urinary tract and the scrotal abscess together formed a “pool” for these pathogen populations. Ceftazidime, meropenem, and moxalactam are excreted mainly via the kidneys, and high concentrations of ceftazidime (2 g intravenous [i.v.], 8,000 to 16,000 mg/L, 0 to 3 h; 110 to 555 mg/L, 6 to 12 h) (19), meropenem (1 g i.v., 45.4 to 1,141.6 mg/L, 0 to 8 h) (20), and moxalactam (1 g i.v., 594 to 2,094 mg/L, 0 to 8 h) (21) that reached the selection window of the *bla*_KPC-2_ multicopy and *bla*_KPC-2_ mutated isolates were detected in the urine, which provides selection pressure for the emergence of resistant isolates.

Taken together, we report the evolutionary trajectory of the KPC-Kp population under clinical antibiotic pressure. The evolution is initiated by an increase in the copy number of *bla*_KPC_ and is then further enhanced by point mutations within the *bla*_KPC_ gene. These findings broaden our understanding of antibiotic resistance development in clinical settings and hence will significantly benefit carbapenem-resistant *Klebsiella pneumoniae* (CRKP) infection treatment.

## MATERIALS AND METHODS

### Strains, ethics, and susceptibility testing.

Strains KPJCL-1 to KPJCL-5 were sequentially isolated from a patient in the ICU. The study was approved by the ethics committee (20170301-3). MICs were determined using broth microdilution (levofloxacin, tigecycline, colistin, and cefiderocol) or agar dilution (imipenem, meropenem, ceftazidime, moxalactam, and CAZ/AVI) according to Clinical and Laboratory Standards Institute (CLSI) standards (22). The MICs of cefiderocol were tested in iron-depleted medium. MICs were interpreted using CLSI breakpoints, where available. The FDA resistant breakpoint of ≥8 mg/L was applied for tigecycline. Escherichia coli ATCC 25922 served as a quality control strain, and the *mcr-1*-positive strain E-FQ (23) served as an extra quality control strain in colistin MIC tests.

### Genomic characterization.

We performed Illumina sequencing (Illumina, San Diego, CA) and long-read sequencing (Oxford Nanopore, Oxford, UK) on the isolates. Sequencing data were hybrid assembled *de novo* using Unicycler (version 0.4.8). Resistance genes and multilocus-sequence typing (MLST) were identified using the CGE server (https://www.genomicepidemiology.org/services/). Gene annotation was performed with RAST (http://rast.nmpdr.org/rast.cgi). Single-nucleotide polymorphism (SNP) typing was performed using Snippy (https://github.com/tseemann/snippy) with the default parameters, and strain KPJCL-2 was used as a reference (whole-genome sequencing data). The SNPs between isolates were calculated by Snp-dists, and the tree was illustrated and annotated using iTOL. Plasmid sequences were compared using the CGView server (http://cgview.ca/).

### Transformation experiment.

The *bla*_KPC-33_ and *bla*_KPC-2_ sequences with their putative promoters were amplified (primers are listed in Table S1 in the supplemental material) and cloned into plasmid pCR2.1 (Invitrogen, Carlsbad, CA, USA). K. pneumoniae ATCC 13883 was used for transformation.

### Bacteria growth assay.

Overnight cultures were diluted 1:100 in cation-adjusted Mueller-Hinton broth (CAMHB) containing a gradient of antibiotic concentrations. Bacterial growth was detected in three replicates using a Bioscreen C MBR machine (Oy Growth Curves Ab Ltd., Finland). Optical density at 600 nm (OD_600_) values of the isolates were compared using a two-sided Mann-Whitney *U* test and are presented as the medians (maximum to minimum values). A *P* value of <0.05 was considered a significant difference. The statistical software used in this study was Prism5.

### Growth competition experiments.

Competition experiments were conducted as described in a previous study (24) in the presence of antibiotic concentrations of significant differences in growth (medium containing ceftazidime [128 mg/L], meropenem [64 mg/L], and moxalactam [512 mg/L] in KPJCL-2 [single copy] and KPJCL-4 [multicopy] growth competition assays and medium containing ceftazidime [512 mg/L] in KPJCL-3 [*bla*_KPC-33_ harboring] and KPJCL-4 growth competition assays). Briefly, KPJCL-2, KPJCL-3, and KPJCL-4 strains were grown overnight at 37°C and were adjusted to the same OD. The adjusted cultures of KPJCL-4 and KPJCL-2 were mixed at a 1:1 or 1:10^3^ ratio (KPJCL-4:KPJCL-2, by decreasing the inoculum of KPJCL-4) in 2 mL of CAMHB or ceftazidime (128 mg/L), meropenem (64 mg/L), and moxalactam (512 mg/L) medium, respectively. The adjusted cultures of KPJCL-3 and KPJCL-4 were mixed at a 1:1 ratio (KPJCL-3:KPJCL-4) in 2 mL of CAMHB or ceftazidime (512 mg/L) medium. The mixed cultures were grown at 37°C for 24 h. The total number of isolates was determined by plating aliquots onto nonselective plates. The numbers of KPJCL-3 and KPJCL-4 colonies were calculated by plating aliquots onto plates containing 32/4 mg/L CAZ/AVI or 512 mg/L moxalactam, respectively. Each ratio of mixed culture was performed in three independent experiments. Competitive advantage was calculated as the competition index (CI), where CI = (isolate A/isolate B)*_t_*_24_/(isolate A/isolate B)*_t_*_0_. The ln (CI) values were compared using a two-sided Mann-Whitney *U* test and are presented as the means ± standard deviation (SD).

### Estimation of the mutation and amplification frequencies.

Overnight KPJCL-2 and KPJCL-4 populations were harvested. The total number of populations was determined by plating serial dilutions on Mueller-Hinton agar (MHA) plates. The *bla*_KPC-2_ G532T mutants in the KPJCL-4 population were selected on plates containing 32 mg/L/4 mg/L CAZ/AVI. The *bla*_KPC-2_ gene of colonies that grew on selective plates was amplified and sequenced to confirm the presence of *bla*_KPC-2_ G532T mutation. The *bla*_KPC-2_ multicopy strains in the KPJCL-2 population were selected through growth on plates containing 512 mg/L moxalactam and were confirmed by quantitative PCR (primers are listed in Table S1). The frequencies were determined by dividing the median number of mutants/multicopy strains by the average number of populations (25).

### Experimental evolution assays.

In KPJCL-2 evolution assays, clones of KPJCL-2 were passaged (1:100) for 6 days in CAMHB or in CAMHB containing ceftazidime (128 mg/L), meropenem (64 mg/L), or moxalactam (512 mg/L) medium or were passaged sequentially in medium containing the three antibiotics each for 2 days. In the KPJCL-4 evolution assay, clones of KPJCL-4 were passaged (1:100) for 6 days in CAMHB and ceftazidime (512 mg/L) medium. Before every transfer, the proportion of subgroups was calculated by dividing the number of colonies growing on plates containing 512 mg/L moxalactam (KPJCL-2 evolution) or 32 mg/L/4 mg/L CAZ/AVI (KPJCL-4 evolution) by the number of total isolates. PCR and quantitative PCR (qPCR) were used to confirm the subgroups.

### qPCR.

2^-ΔΔ*C^T^*^ method was used for relative quantitation of the *bla*_KPC_ gene normalized to the plasmid replication protein gene (*rep*) or *rep* normalized to the *pgi* housekeeping gene (primers are listed in Table S1). The mean *C_T_* value was calculated from three replicate reactions, and the ΔΔ*C_T_* value was calculated from three different DNA preparations. Data are presented as the means ± SD.

### Data availability.

The sequencing data have been deposited in GenBank under the accession numbers JAKJSA000000000, JAKJRZ000000000, JAKJRY000000000, JAKJRX000000000, and JAKJRW000000000.
