# Supplementary material for: The Effect of β-Lactam Antibiotics on the Evolution of Ceftazidime/Avibactam and Cefiderocol Resistance in KPC-Producing Klebsiella pneumoniae
Source: Antimicrob Agents Chemother. 2023 Feb 16;67(3):e01279-22. doi: 10.1128/aac.01279-22 (PMC10019305; doi:10.1128/aac.01279-22)

Table S1. Primers used in this study.

Name of primers	Sequence	Description
KPC-N10-F	GCTCCACCTTCAAACAAGGA	confirming bla_{KPC-2} mutation
KPC-891-R	GGTGGGCCAATAGATGATTT	confirming bla_{KPC-2} mutation
TraI-F	TAAAATGGCAGAGAACAAACCTGAC	confirming bla_{KPC-2} mutation regions
TraI-R	GCTCCACCTTCAAACAAGGA	confirming bla_{KPC-2} mutation regions
IS-F	TGTAACCTGTGGAGGTCTGAATAAG	confirming bla_{KPC-2} mutation regions
IS-R	GCTCCACCTTCAAACAAGGA	confirming bla_{KPC-2} mutation regions
Tandem-F	CTCTACAGAAAACCGGTCAC	confirming bla_{KPC-2} mutation regions
Tandem-R	CAAATCGGGATCATGCAGAA	confirming bla_{KPC-2} mutation regions
PGI-F	TTCATCGCTCCGGCTATCAC	real-time PCR
PGI-R	CCGGGTCTTTACCCTGATCG	real-time PCR
REP-F	ACAGGGAACGTCAGGATATT	real-time PCR
REP-R	TTACGTGACAGAATCATGCG	real-time PCR
KPC-F	GCGGCAGCAGTTTGTTGATT	real-time PCR
KPC-R	CGGCATAGTCATTTGCCGTG	real-time PCR
KPC-promoter-F	TCTACAACCACAGCATTCCG	TA-cloning
KPC-promoter-R	TACAGAAAACCGGTCACACG	TA-cloning

Table S2. Effect of bla_{KPC-2} G532T mutation on MICs change of CAZ/AVI and cefiderocol.

Isolates	IMP	CAZ/AVI	CAZ	FEP	MOX	CRO	CFDC
KPJCL-3	128	>128	4096	2048	2048	>256	>32
KPJCL-4	128	8	1024	2048	>2048	>256	4
ATCC 13883	1	0.25	0.25	0.25	0.25	0.06	0.25
ATCC 13883::pCR2.1	1	0.5	0.5	0.5	0.5	0.125	0.25
ATCC 13883::pCR2.1-bla_{KPC-33}	0.5	32	>128	8	4	8	8
ATCC 13883::pCR2.1-bla_{KPC-2}	16	1	16	64	8	>128	1
ATCC 25922	0.25	0.25	0.25	0.125	0.25	0.06	0.125

IMP: Imipenem CAZ/AVI: Ceftazidime/avibactam CAZ: Ceftazidime FEP: cefepime MOX: Moxalactam CRO: Ceftriaxone CFDC: Cefiderocol.

Table S3. Characteristics of ***bla*****$_{KPC-2}$ multi-copy subgroups from** ***in vitro*** **evolution experiment.**

Isolate(s)	Relative plasmid copy number	Relative bla_{KPC-2} copy number	MIC (mg/L)				
			CAZ/AVI	CFDC	MEM	CAZ	MOX
KPJCL-2	1.003±0.035	0.998±0.019	4	4	128	128	512
KPCAZ-E	1.248±0.215	4.750±0.501	16	8	512	512	2048
KPMEM-E	0.967±0.124	5.089±0.860	16	8	512	512	2048
KPMOX-E	0.933±0.100	5.598±0.373	16	8	512	512	2048
KPMulti-E	1.138±0.357	5.615±0.681	16	4	512	512	2048

KPJCL-4	1.150±0.131	3.067±0.220	8	4	512	1024	4096
KPJCL-3	1.163±0.067	2.220±0.036	256	>32	256	4096	2048

CAZ/AVI: Ceftazidime/avibactam CFDC: Cefiderocol CAZ: Ceftazidime FEP: cefepime MOX: Moxalactam.

Figure S1. Clinical timeline of the isolates.

Isolation of bacterial strains and history of antibiotics use. Bars represent the antimicrobial treatment and duration. The flags show the name and isolation time of the isolates. CAZ/AVI: ceftazidime/avibactam, CFDC: cefiderocol, MEM: meropenem, LEV: levofloxacin, MOX: moxalactam, CAZ: ceftazidime, AMC: amoxicillin/clavulanic acid, SCF: cefoperazone/sulbactam.

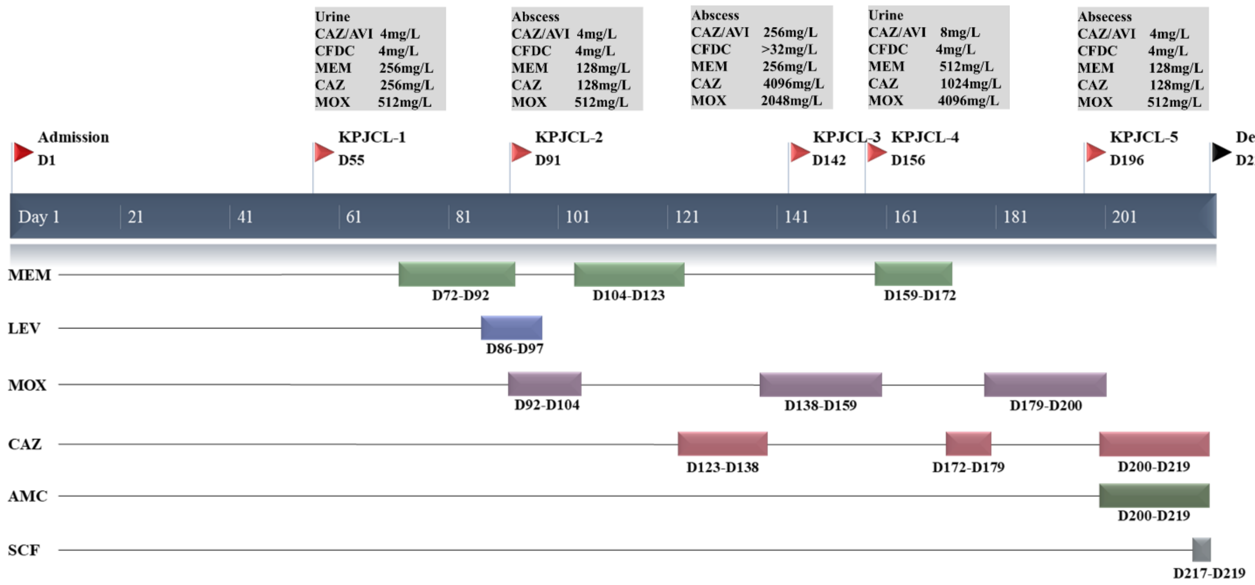

Figure S2. Genotypic characterization of isolates.

a, The SNP numbers detected between each strain. Comparative analyse shows high homology among the isolates. **b**, Maximum-likelihood phylogenetic tree. The 5 isolates are divided into two groups. The isolates in pink area contain plasmids with single-copy of *bla*$_{KPC-2}$ gene and the isolates in blue area contain plasmids with multi-copy of *bla*$_{KPC-2}$ genes. The phylogenetic tree provides clues of the *in vivo* evolution of the series isolates. KPJCL-2 was used as the reference strain.

a

	KPJCL-1	KPJCL-2	KPJCL-3	KPJCL-4	KPJCL-5
KPJCL-1	0				
KPJCL-2	5	0			
KPJCL-3	6	7	0		
KPJCL-4	5	6	3	0	
KPJCL-5	4	5	6	5	0

b

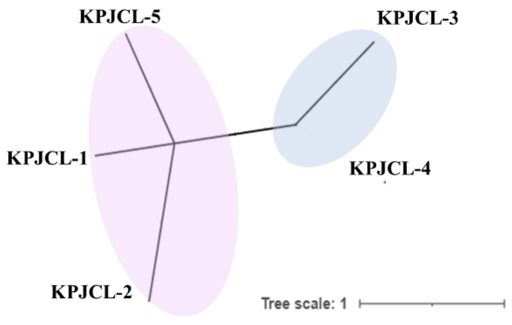

Figure S3. Growth difference of KPJCL-2 and KPJCL-4 under ceftazidime concentrations.

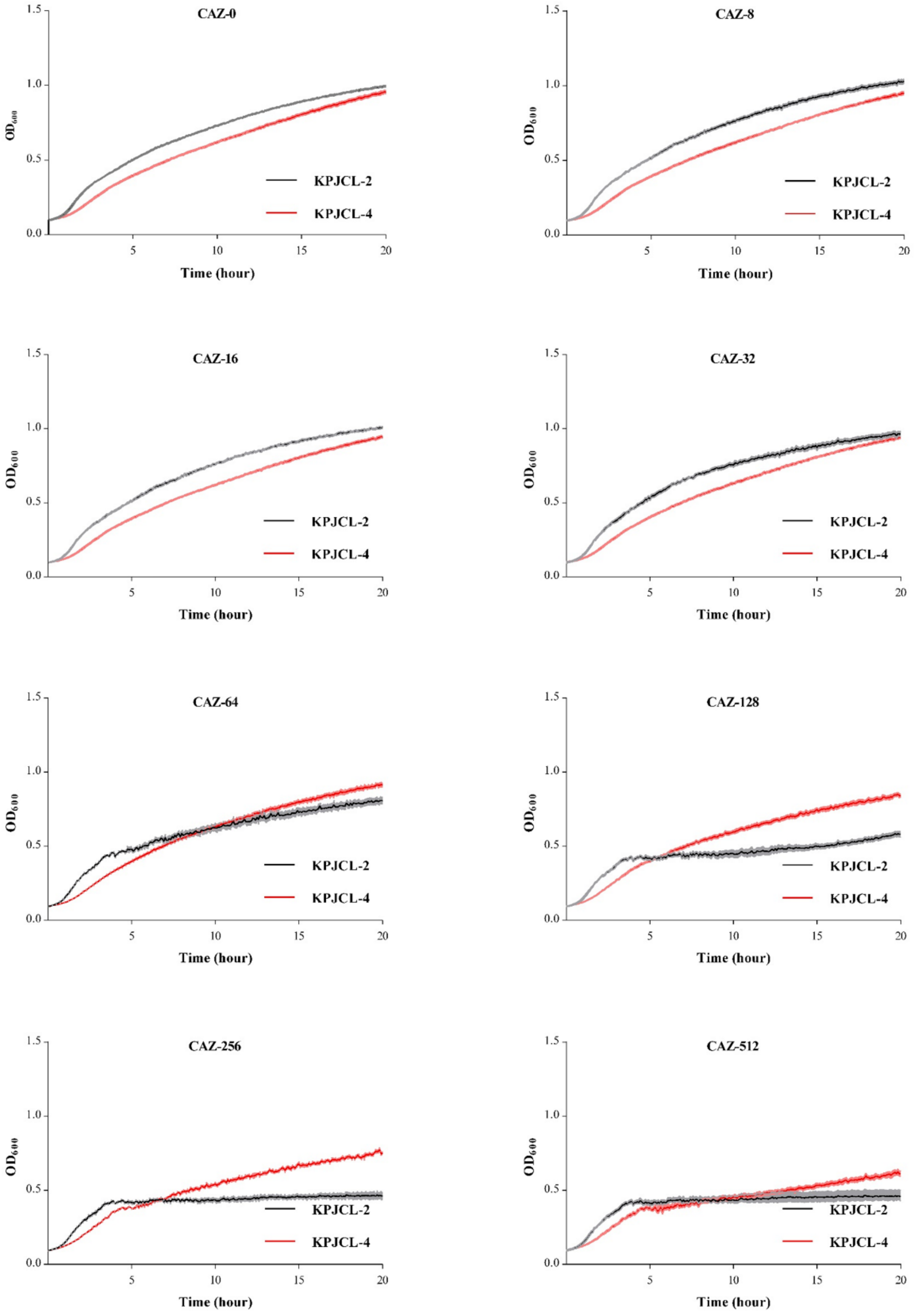

Figure S4. Growth difference of KPJCL-2 and KPJCL-4 under meropenem concentrations.

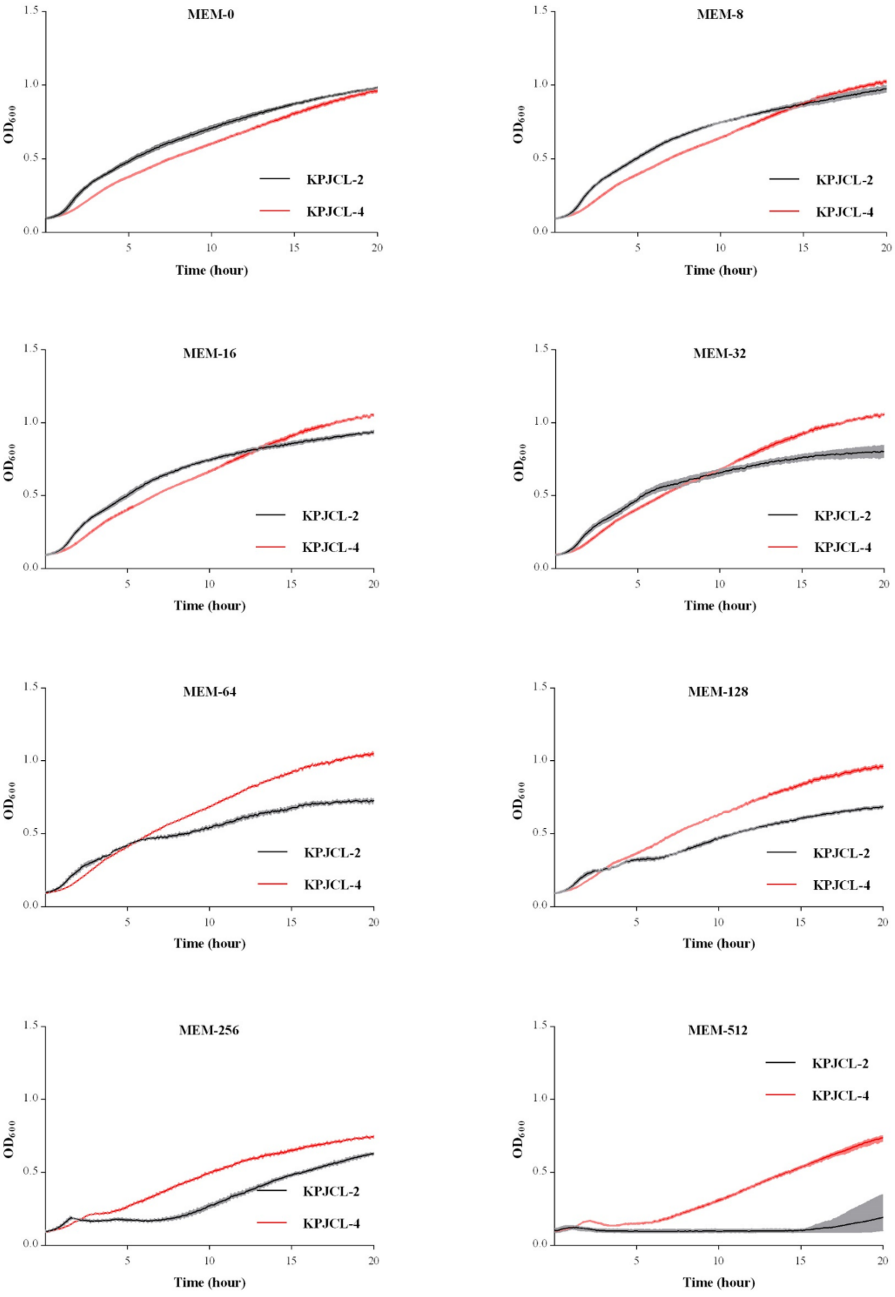

Figure S5. Growth difference of KPJCL-2 and KPJCL-4 under moxalactam concentrations.

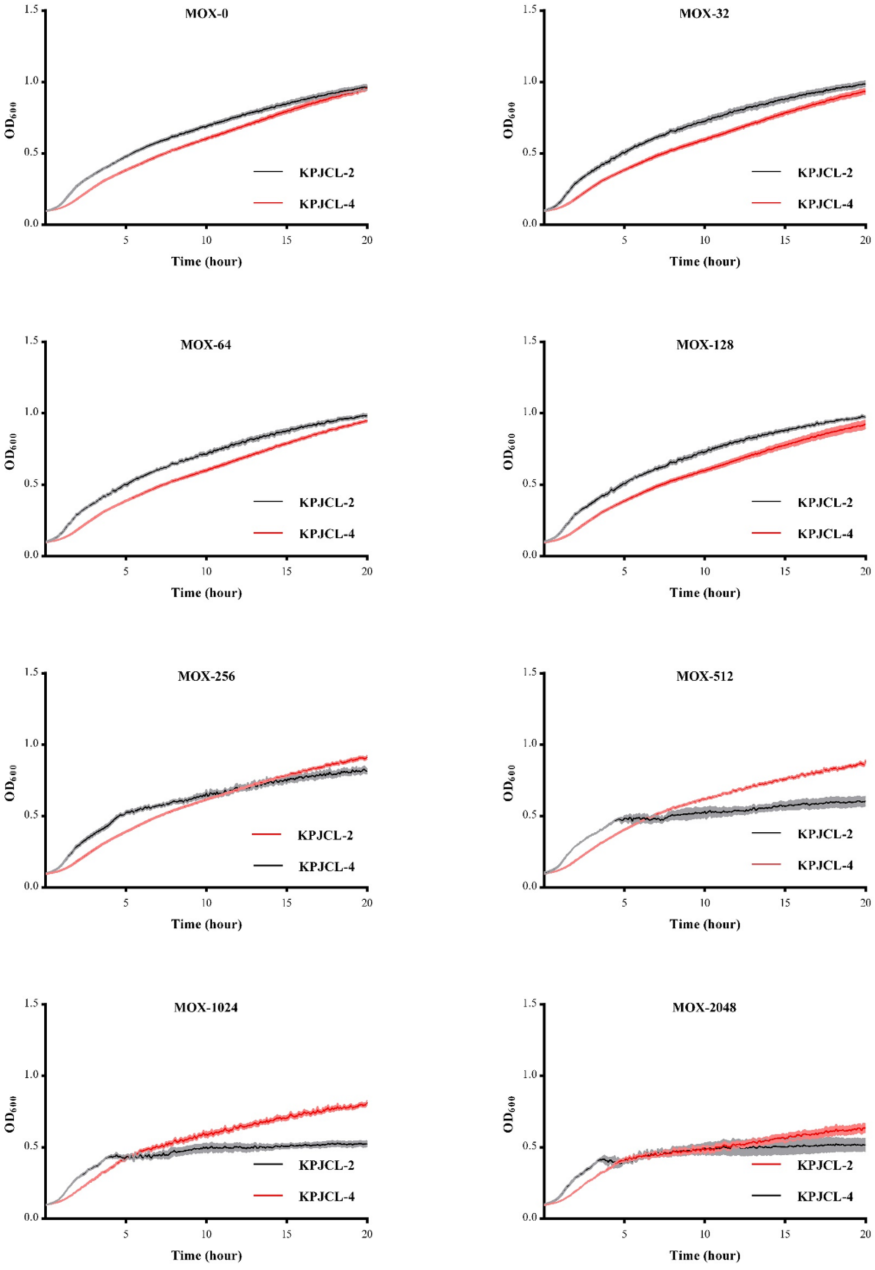

Figure S6. Growth difference of KPJCL-3 and KPJCL-4 under ceftazidime concentrations.

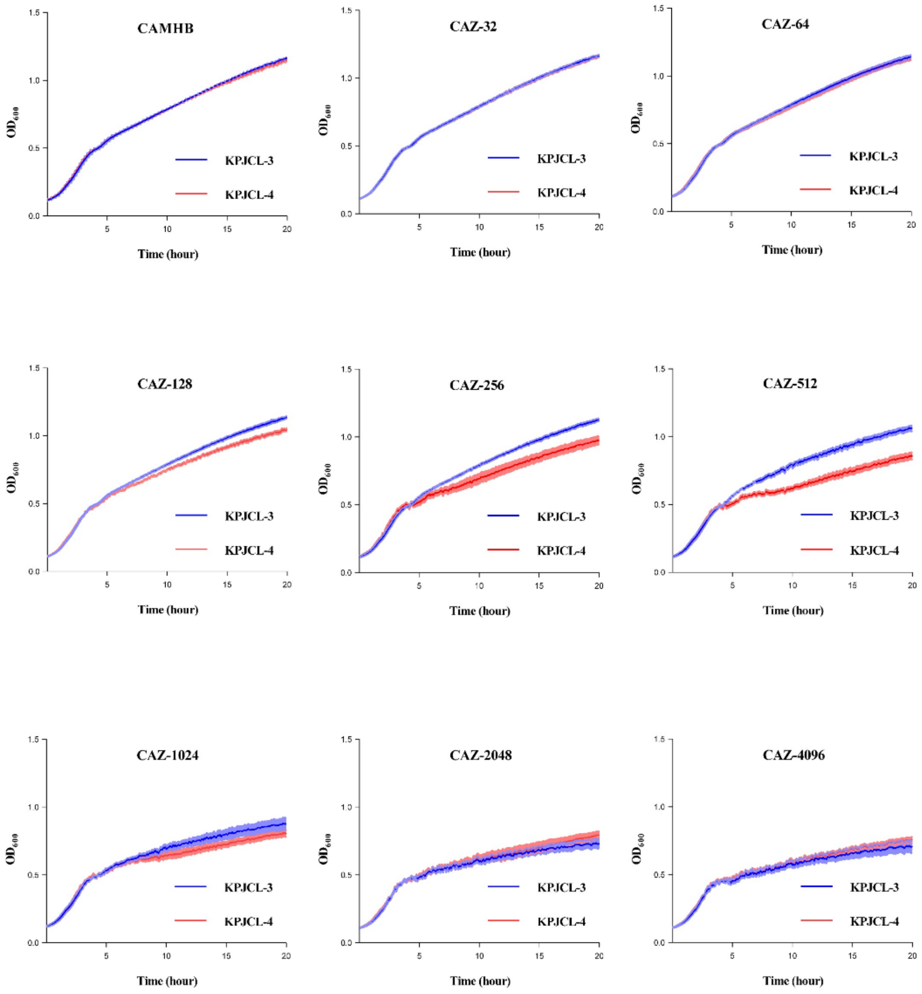

Figure S7. Amplification and mutation frequency.

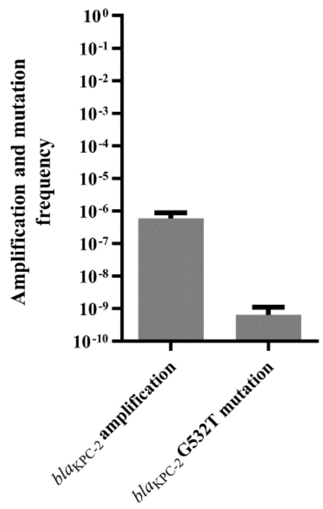

Figure S8. Evolution of KPC-Kp population under clinical antibiotic pressure. In the first step, *bla*$_{KPC-2}$ multi-copy strains (KPJCL-4 as the representative strain) was screened out from the *bla*$_{KPC-2}$ single-copy population (KPJCL-2 as the representative strain) under the pressure of ceftazidime, meropenem, or moxalactam, which contributes to the elevated CAZ/AVI MIC. In the second step, the population further evolved to high-level CAZ/AVI resistance and reduced cefiderocol susceptibility by the enrichment of *bla*$_{KPC-2}$ mutation strains (KPJCL-3 as the representative strain) under ceftazidime selection.

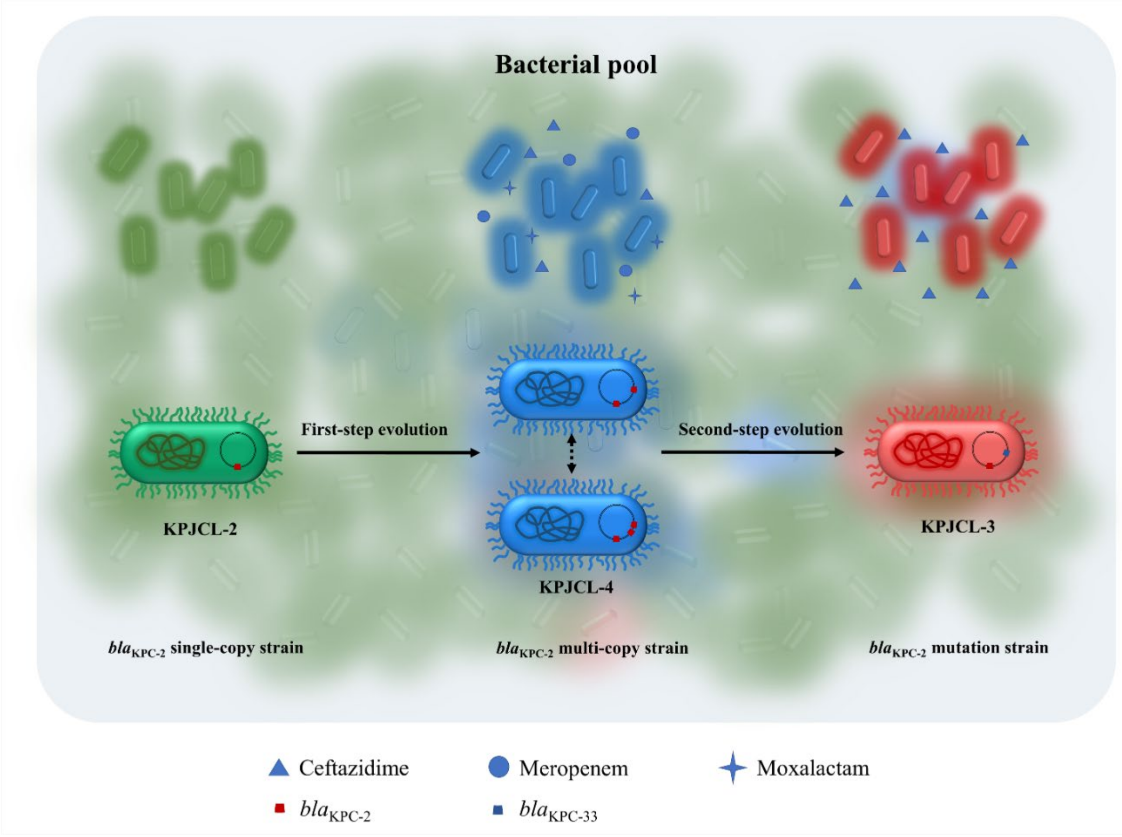

Supplement: Supplemental file 1 — Supplemental material. Download aac.01279-22-s0001.pdf, PDF file, 1.7 MB [file aac.01279-22-s0001.pdf]
